# Role of machine and organizational structure in science

**DOI:** 10.1371/journal.pone.0272280

**Published:** 2022-08-11

**Authors:** Moe Kyaw Thu, Shotaro Beppu, Masaru Yarime, Sotaro Shibayama

**Affiliations:** 1 Graduate School of Public Policy, The University of Tokyo, Tokyo, Japan; 2 School of Engineering, The University of Tokyo, Tokyo, Japan; 3 Division of Public Policy, The Hong Kong University of Science and Technology, Kowloon, Hong Kong; 4 Institute of Future Initiatives, The University of Tokyo, Tokyo, Japan; 5 School of Economics and Management, Lund University, Lund, Sweden; Universita degli Studi di Foggia, ITALY

## Abstract

The progress of science increasingly relies on machine learning (ML) and machines work alongside humans in various domains of science. This study investigates the team structure of ML-related projects and analyzes the contribution of ML to scientific knowledge production under different team structure, drawing on bibliometric analyses of 25,000 scientific publications in various disciplines. Our regression analyses suggest that (1) interdisciplinary collaboration between domain scientists and computer scientists as well as the engagement of interdisciplinary individuals who have expertise in both domain and computer sciences are common in ML-related projects; (2) the engagement of interdisciplinary individuals seem more important in achieving high impact and novel discoveries, especially when a project employs computational and domain approaches interdependently; and (3) the contribution of ML and its implication to team structure depend on the depth of ML.

## Introduction

Scientific knowledge shapes the foundation of the modern society, contributing to economic, social, and technological progress [1, 2]. The progress of science relies on various technical bases such as experimental techniques [3]. Among others, computational techniques play a crucial role in various parts of scientific research [4, 5], and their role has been becoming more fundamental especially with the advancement of artificial intelligence, or more specifically machine learning (ML) [6].

Increasing examples have been reported in various domains, in which machines work alongside humans to push forward the progress of science. For example, in life sciences, protein-protein interactions are predicted to understand disease mechanisms [7]; in chemistry, optimal chemical reaction paths are predicted [8]; and in material sciences, physical properties of new materials are predicted [9]. These examples are characterized by ML, in which well-trained algorithms engage in complex tasks and directly contribute to making discoveries but not only merely automating the work process.

As contemporary science is usually based on a team activity [10], the integration of machines as creative agents can influence the optimal design of work and organizations [11–13]. While the interaction between human and machine has been studied at a micro (cognitive) level [4] or a at a macro level [6], the literature has been rather silent as to the role of machine in organization design [14]. Though a few studies described the patterns of collaboration (e.g., international vs. domestic collaboration) in ML-related projects [15, 16], no previous study to the best of our knowledge has investigated the impact of ML on the internal organizational design of scientist teams. This study thus aims to investigate the team structure of ML-related projects and analyze the contribution of ML to scientific knowledge production under different team structure.

To this end, we draw on bibliometric analyses. Our primary interest is how computational science techniques are integrated into the fields of conventional domains of science ("*domain science*" hereafter). Namely, our analysis includes six domains–agriculture, biology, chemistry, material sciences, medicine, and physics. To highlight the role of machines, we exploit a comparative approach, contrasting (1) ML-related projects (combination of computational and domain sciences) and (2) ML-unrelated projects (pure domain sciences). We collected approximately 2,500 ML-related and 22,000 ML-unrelated scholarly publications. With bibliometric and text analyses, we operationalized key variables of our interest. We investigated the quality (citation *impact* and *novelty*) of publication output produced by research teams with different team characteristics. Our results suggest (1) that interdisciplinary collaboration between domain scientists and computer scientists as well as the engagement of interdisciplinary individuals who have expertise in both domain and computer sciences are common in ML-related projects, (2) that the engagement of interdisciplinary individuals seem more important in achieving high impact and novel discoveries, especially when a project employs computational and domain approaches interdependently, and (3) that the contribution of ML and its implication to team structure depend on the depth of ML, in particular deep learning being associated with greater impact but with lower novelty.

This paper is structured as follows. The next section reviews literature on the use of ML and on the organizational design of science and formulates a few hypotheses. The following section outlines the method and data. Then, the results from bibliometric analyses are presented. The final section discusses the results and concludes.

## Theory and hypothesis

### Role of machine in science

Though the use of ML in science has substantially grown in the 2010s [6], computational techniques have long been playing critical roles in science [17]. In empirically driven domains of science such as physics and biology, statistical approaches have been actively used [18], and enhanced computational power contributed to the progress of these fields [19]. Further, data have been accumulated for collective use in various fields (e.g., genome data in life sciences, material data in materials science), and access to large-scale data facilitated data-driven approaches in these fields [20]. These technical bases coupled with algorithmic breakthrough in the 2010s have transformed ML into a practical tool [21, 22]. ML has been applied to tackle broad areas of problems from industry to academia, including autonomous driving, robotics, communications, manufacturing, and medical diagnosis [23–26].

The technical core of ML is a model based on neural network, decision trees, and so forth [27]. A model is trained by data and then applied to additional data to make predictions. In academic research, domain scientists exploit this prediction capability to predict scientific laws of their interest, such as optimal chemical reaction paths, physical properties of materials, and protein-protein interactions [7–9].

Empirical research usually involves iterated cycles of hypothesis formulation, data collection, and data analysis [28, 29]. Domain scientists can incorporate ML into different parts or stages of this process. For example, ML can be used in later stages–data are generated through non-computational approaches (e.g., experiment) and fed into ML. In this case, the output of ML may become findings reported in publications. Alternatively, ML can be used in earlier stages–a hypothesis is formulated based on machine prediction and is tested by non-computational approaches. In this case, the role of ML is more exploratory and its output may be less explicitly presented in publications. Finally, ML may be used for data collection to automate the process of collecting, cleaning, and coding the data. In such cases, the use of ML improves the efficiency of scientific research but its role may be less apparent in publications.

Only a few studies have investigated the impact of ML on scientific knowledge production [30], but we argue that ML can bring various values depending on how it is incorporated into a research process. At the most primitive level, ML may make scientific research more efficient and productive, for example, when ML is used for automation. ML is also expected to improve the quality of information extracted from the data. By selecting a right model and carefully tuning it, scientists may be able to extract more accurate or precise information than simpler statistical approaches can do. Finally, most fundamentally, ML may help domain scientists reach a discovery beyond their cognitive capacity. As a result of rapid progress of science, fully mastering domain knowledge has become a challenge for human scientists [31]. The increasing specialization of science also has made it challenging to integrate knowledge in multiple domains, even though such is an important route for scientific discoveries [32]. With the computer processing power, ML may help overcome these challenges attributed to the limit of human cognition.

### Organization of science and role of machine

For fulfilling these values of ML in domain science, the expertise in computational science and that in domain science need to be integrated. ML algorithms may be able to improve themselves automatically through the use of data [33], and this autonomous nature makes machines creative agents. Yet, machines require a substantial care by human scientists, who develop, run, and assess a model as well as interpret the output generated by the model [4, 22]. This process requires both the knowledge of domain science and that of computer science. In fact, a bibliometric analysis found that the vast majority of ML-based research involves collaboration [15], implying the integration of two sets of expertise is a key to success.

The application of ML for domain science can be considered a case of interdisciplinary research [34, 35]. Previous studies discussed various challenges associated with interdisciplinary research [36, 37]. The studies consistently suggested organizational challenges for example in the coordination of tasks and in communication between diverse scientists, as well as in trust building which is critical to share insights and research findings among members. In applying computational techniques to other domains, the same organizational challenges have been suggested [38–40].

Scientist teams set up various organizational arrangements to overcome such challenges. One potential solution concerns the proximity of collocation. Previous studies evaluated the impact of the proximity of team members on team performance in various contexts, by and large suggesting that proximity facilitates communication and thus performance [41]. In the context of science, collaboration may occur in proximity (e.g., intra-organizational collaboration) or remotely (e.g., inter-organizational collaboration) [15, 42]. As an extreme case of proximity in science, collaboration can occur within a lab. A lab is an organizational unit more permanent than a project team, and it offers the organizational basis for scientific activities in traditional university systems [28, 29, 43]. Recent years have seen interdisciplinary labs being formed so that multiple disciplines can interact effectively [44, 45]. Apparently, effective and dense communication is expected among lab members who share the workspace on a daily basis. For example, members can monitor one another, which may allow a member to detect a problem that another member struggles with and quickly find a solution to it [29, 46].

Hypothesis 1 (H1): *The proximity of collaboration between computer scientists and domain scientists is positively associated with the quality of research output*.

As another route to tackle interdisciplinary research challenges, individual scientists may acquire expertise in multiple domains. If an individual scientist has skills both in computer science and in domain science, the aforementioned organizational challenges can be resolved within him/herself. In fact, some areas of domain science recognized the promising power of computational techniques and actively incorporated computational science, such as bioinformatics in the biology domain [47]. These domains tend to offer a curriculum to train for computational techniques, which systematically develops interdisciplinary scientists at the intersection of domain and computer sciences. This is becoming common, as computational techniques are increasingly available from external sources such as publicly shared codes and commands implemented in software, and thus, the skill requirement on computer science may be lowered.

Such interdisciplinary individuals can also play a boundary spanner role, who mediates members of different expertise [48, 49]. They translate the languages of domain scientists and computer scientists and facilitate their integration. Thus, the organizational challenges in interdisciplinary research are alleviated, which helps achieve expected interdisciplinary output [50].

Hypothesis 2 (H2): *The engagement of an interdisciplinary scientist who has expertise in both computer science and domain science is positively associated with the quality of research output*.

Among these organizational arrangements, the optimal form may depend on how computer science expertise is applied to domain science, or on the interdependency of the two areas of tasks [51]. On the one hand, task interdependency can be high if the output of domain science tasks is used as the input of computer science tasks, or vice versa. The two areas of tasks may be repeated in a cyclic way. In such a scenario with high task interdependency, organizational arrangement for interdisciplinary integration is expected to be more important. On the other hand, task interdependency can be low if the two areas of tasks are modularized. For example, computer scientists may apply their ML models to publicly available data from domain sciences. Domain scientists may use ML only for data preparation (cleaning, etc.) or may use established ML algorithms. In these cases, the interface between the two areas of tasks is minimized, and thus, the above discussed organizational arrangement becomes less relevant.

Hypothesis 3A (H3A): *The proximity of collaboration between computer scientists and domain scientists is more positively associated with the quality of research output when the computer-related tasks and domain tasks are interdependent*.
Hypothesis 3B (H3B): *The engagement of an interdisciplinary scientist who has expertise in both computer science and domain science is more positively associated with the quality of research output when the computer-related tasks and domain tasks are interdependent*.

## Methods and data

### Data

To test our hypotheses, we draw on bibliometric data collected from the Web of Science (WoS). Our primary interest is in the integration of computational science (ML) and domain science. To highlight the contribution of ML, we draw on a comparative approach, contrasting (1) ML-related projects (combination of computation and domain sciences) and (2) ML-unrelated projects (purely domain science). The unit of analysis is a project team, which is operationalized by a group of authors of a publication.

We employ the following sampling strategy. First, we chose six domains–agriculture, biology, chemistry, material sciences, medicine, and physics. The selection of these domains is based on WoS Subject Categories (SC). We chose 20 SCs in total within these domains (see S1 Appendix).

Second, in these domains (SCs), we aimed to choose journals that are as mono-disciplinary as possible for two reasons. First, this is to lower the risk of sampling ML-related projects that are unrelated to domain science. We assume that mono-disciplinary journals set a clear scope of publication, with such a risk being mitigated. Second, to clarify the impact due to the integration of computer science and domain science, we minimized noise stemming from interdisciplinarity within a domain. To these ends, we chose up to five journals in each SC that are associated with a single SC (not associated with any other SC).

Third, in these journals we selected two sets of papers, ML-related and ML-unrelated. We first searched for ML-related papers with "machine learning", "deep learning", and "artificial intelligence" as search keywords, which resulted in 2,500 papers.

Next, we collected ML-unrelated papers that include none of the ML-related keywords. For clearer comparison, for each ML-related paper, we randomly selected up to 10 ML-unrelated papers published in the same journal and in the same year. We found 22,300 ML-unrelated papers. In total, we sampled 25,000 papers with 10% of ML-related papers and downloaded their bibliometric information.

### Measures

#### Quality of research output

As the dependent variables of our analyses, we prepared two measures of scientific quality. First, we use the citation count as of 2021 to assess the impact of the findings reported in the paper. To mitigate the skewness, we took a natural logarithm of citation count (*Impact*).

Second, we measure the novelty of a paper. This is because we are interested in to what extent ML contributes to creating new knowledge beyond human cognition. We drew on the recombinant novelty concept [32, 52] and followed the operationalization by Matsumoto, et al [53]. The method considers a paper to be novel when it cites a pair of references that have rarely been cited together before. For easier interpretation we transformed the measure into a rank measure so that its values are uniformly distributed between 0 and 1, with 0 being the least novel and 1 being the most novel (*Novelty*). We computed this variable only for part of our sample (67%) due to lack of access to citation network information.

#### Computer-domain collaboration

Since we are interested in the integration of computational expertise and conventional expertise, we investigated the forms of collaboration. To this end, we scrutinized the names of authors’ affiliated organizations to distinguish if an organization has a computational background or not. Then, we consider an organization to be computational if the name includes "computation", "information", or "system", and we consider an organization to be in a traditional domain if the name includes none of them. Using this distinction of organizations, we prepared a few variables. We first measured if a team involved both computational and domain organizations (instead of involving only domain organizations). A dummy variable is coded 1 if at least one affiliated organization is computational and 0 otherwise (*Comp-Domain Collab*).

#### Proximity of collaboration

To further investigate the proximity in collaborating parties, we examined whether a team involved computational and domain organizations inside the same parent organization (i.e., a computational department and a domain department in the same university). In such teams, a dummy variable is coded 1 and otherwise 0 (*Intra-Org Collab*). Similarly, if a team involved computational and domain organizations in two different organizations, another dummy variable is coded 1 and otherwise 0 (*Inter-Org Collab*). Note that one team can involve both intra-organizational collaboration and inter-organizational collaboration.

#### Interdisciplinary individuals

Third, we measured whether an individual team member has both computational and domain expertise in two ways. One measure is based on organizational affiliation. If an individual member of a team (an author of a paper) is affiliated with both computational and domain organizations, we consider that the member has both computational and conventional expertise and plays a boundary spanner role at the individual level. A dummy variable is coded 1 if a team has at least one author affiliated with both types of organizations and 0 otherwise (*Multi-Affiliation*). The other measure is based on the previous experience of individual members. For feasibility, we focused on the corresponding author of each paper and tracked WoS Subject Categories (SCs) associated with their previous publications. We grouped SCs into computer-related and domain-related SCs, and coded a dummy variable 1 if the previous paper is associated with both a computer-related SC and a domain-related SC, and 0 otherwise (*Multi-Expertise*).

#### Interdependency of computer and domain science

ML may be used in different ways in domain sciences. We sampled ML-related papers and categorized them into two groups by reading the method section of the papers. The first group of papers integrates both computational approaches and domain approaches (e.g., experiment, observation), whereas the second group uses mainly computational approaches, typically based on secondary data. We assume that this is a critical distinction in that the former group (*Computer-Domain Integrated*) should require a greater extent of integration between computer and domain expertise compared with the latter group (*Computation-Focused*).

#### ML-related project

Part of the following analyses compare ML-related papers and ML-unrelated papers. For this comparison, we prepared a dummy variable, coded 1 if a paper is ML-related (i.e., including "machine learning", "deep learning", "artificial intelligence" in the title, abstract, or keywords) and 0 otherwise (*ML-related*).

#### Depth of ML

ML can mean various technologies. In fact, common technical keywords in our selected papers include, for example, "neural network", "classification", "regression", "support vector machine", and "random forest." Some of these have been used traditionally (e.g., regression analyses). To highlight the impact of machines, we attempted to differentiate the depth of models on which ML is carried out. Hence, we aim to contrast "deep" learning (DL) and "non-deep" learning (non-DL). This is because the distinction should affect the computer-science expertise required in a team and because deeper learning might provide new values for science beyond what traditional statistical techniques can do. Making this technical distinction based on text information is challenging because the detail of ML models is not always available. For feasibility, we consider that a project involved DL if a paper includes "deep learning" or "neural network" in the abstract or in the keywords (*DL-related*).

#### Control variables

In the regression analyses, we control for several variables. As the base characteristic of a team, we counted the number of authors of each paper (*#Author*), the number of organizations (university, firms, etc.) included in the author address (*#Org*), and the number of countries included in the author address (*#Country*). We also control for university-industry collaboration as it is known to affect scientific performance [54]. A dummy variable is coded 1 if a paper has both academic and industrial affiliations and 0 otherwise (*Univ-Industry Collab*). We include publication year dummies as well as journal dummies. In so doing we control for systematic differences in the quality measures between publication years and between disciplines.

## Results

### Description of team structure

We first describe our dataset. Table 1 presents the descriptive statistics and correlation matrix of all the variables. The publication of ML-related papers has recently grown substantially, and 90% of our sampled papers were published in the last five years (2017–2021). In terms of scientific domains, the ML-related papers are in Agriculture (7%), Biology (31%), Chemistry (9%), Material sciences (5%), Medicine (25%), and Physics (24%). The authors of the ML-related papers spread across many countries, but major countries include the US (34%), China (23%), the UK (9%), and Germany (9%). Finally, we examined the technical keywords common in the ML-related papers, finding "neural network" (28%), "classification" (28%), "regression" (12%), "support vector machine" (11%), and "random forest" (9%) among others.

**Table 1:** Descriptive statistics and correlation matrix.

	Variables	Mean	S.D.	Min	Max	1	2	3	4	5	6	7	8	9	10	11	12
1	Impact	1.010	1.173	.000	8.218												
2	Novelty	.500	.289	.000	1.000	-.053											
3	Ln(#Author)	1.715	.670	.000	5.198	.077	.071										
4	Ln(#Org)	1.101	.727	.000	5.176	.071	.049	.668									
5	Ln(#Country)	.296	.460	.000	3.296	.095	.008	.327	.515								
6	Univ-Industry Collab	.064	.246	.000	1.000	.033	-.030	.167	.197	.136							
7	Comp-Domain Collab	.174	.379	.000	1.000	.070	.011	.105	.256	.152	.062						
8	Intra-Org Collab	.084	.277	.000	1.000	.064	.023	.103	.212	.045	.018	.660					
9	Inter-Org Collab	.154	.361	.000	1.000	.064	.010	.124	.274	.193	.079	.932	.515				
10	Multi-Affiliation	.100	.300	.000	1.000	.071	.019	.075	.226	.131	.049	.727	.582	.684			
11	Multi-Expertise	.151	.358	.000	1.000	.017	-.018	-.131	-.044	.015	.004	.227	.142	.210	.161		
12	ML-related	.103	.304	.000	1.000	.095	.004	-.032	.009	-.010	.086	.192	.134	.171	.130	.231	
13	DL-related	.040	.196	.000	1.000	.046	-.021	-.033	-.020	-.014	.055	.130	.082	.117	.086	.156	.602

Note. N = 24,641.

#### Team size

**Fig 1:**
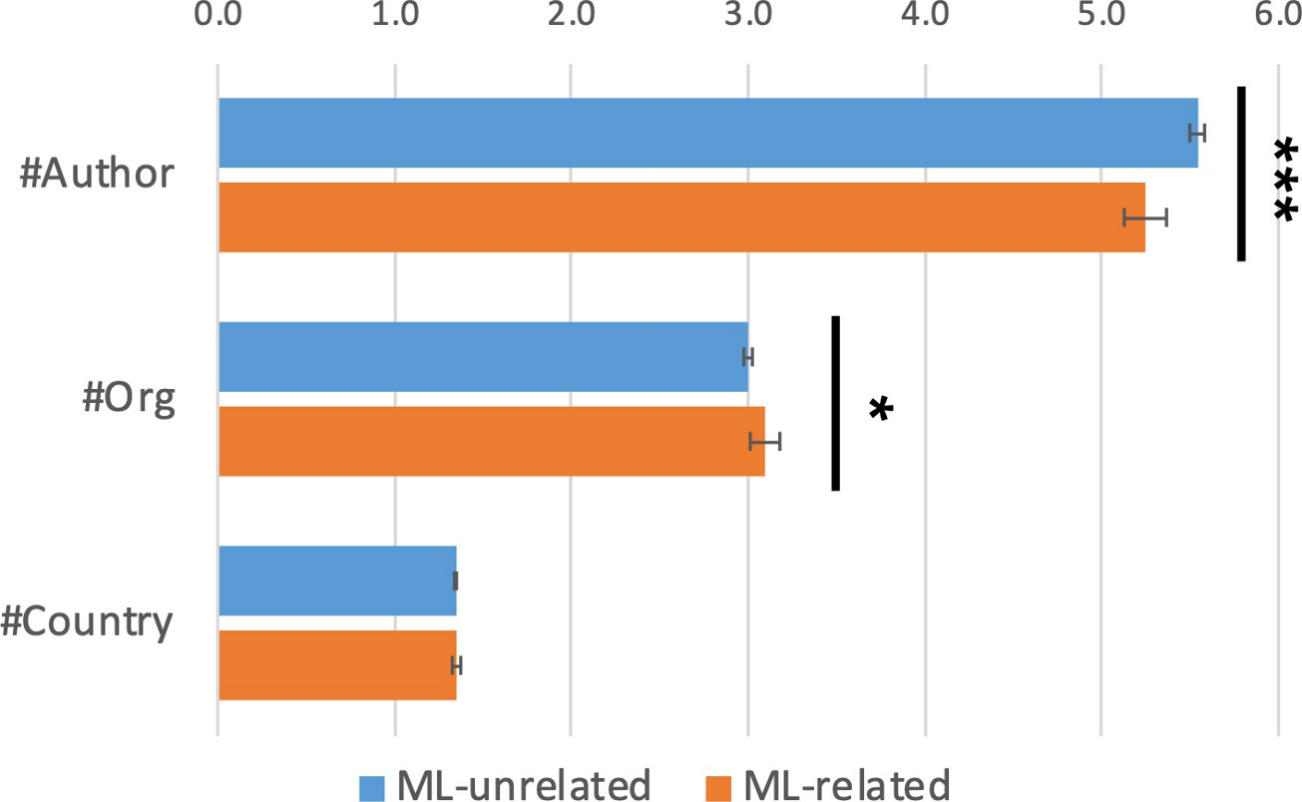
Team size. Team size is estimated by ordinary least squares (OLS) regressions controlling for publication years and journals. The error bars indicate one standard error. Two-tailed test: *p<0.05, ***p<0.001.

We then describe the organizational features of ML-related projects as opposed to ML-unrelated projects. Fig 1 first shows the team size of ML-related and ML-unrelated projects. We regressed the size variables on *ML-related* and other control variables. The top bars indicate that ML-related projects involve slightly fewer authors than ML-unrelated projects (5.3 vs. 5.5, p < .001). This may be because ML-related projects require less physical work and thus fewer members. The second bars, however, indicate that ML-related projects involve more organizations (3.1 vs. 3.0, p < .05) probably because the ML-related projects tend to require a broader set of expertise (i.e., computational and domain). Finally, the bottom bars show no significant difference in the number of involved countries.

#### Computer-domain collaboration

**Fig 2:**
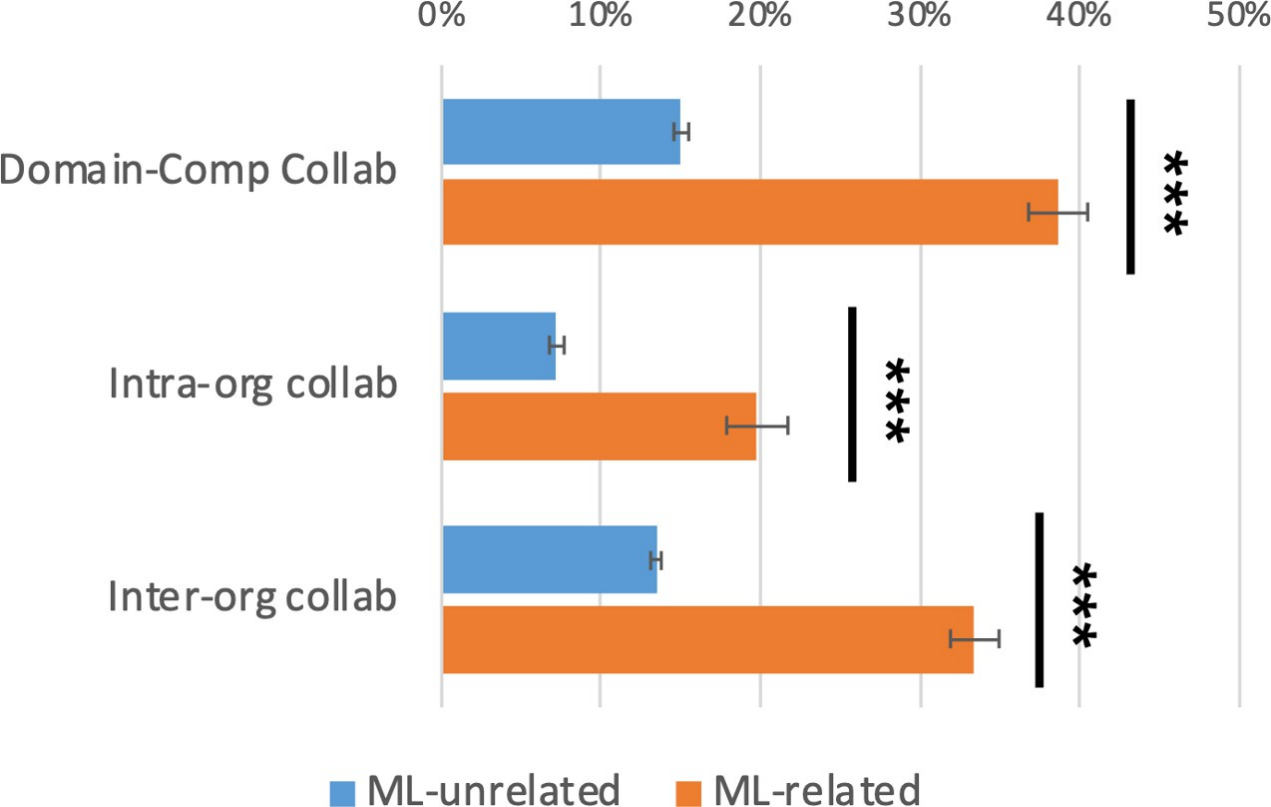
Collaboration form. Collaboration forms are estimated by logit regressions controlling for publication years and journals. The error bars indicate one standard error. Two-tailed test: ***p<0.001.

Then, we analyze the collaboration forms between computational and conventional organizations (Fig 2). As expected, domain-computer collaboration is more common in ML-related projects than in ML-unrelated projects (39% vs. 15%, p < .001). Domain-computer collaboration is broken down into intra-organizational and inter-organizational collaborations, both of which are more common in ML-related projects (20% vs. 7%, p < .001 and 33% vs. 14%, p < .001).

#### Interdisciplinary individuals

**Fig 3:**
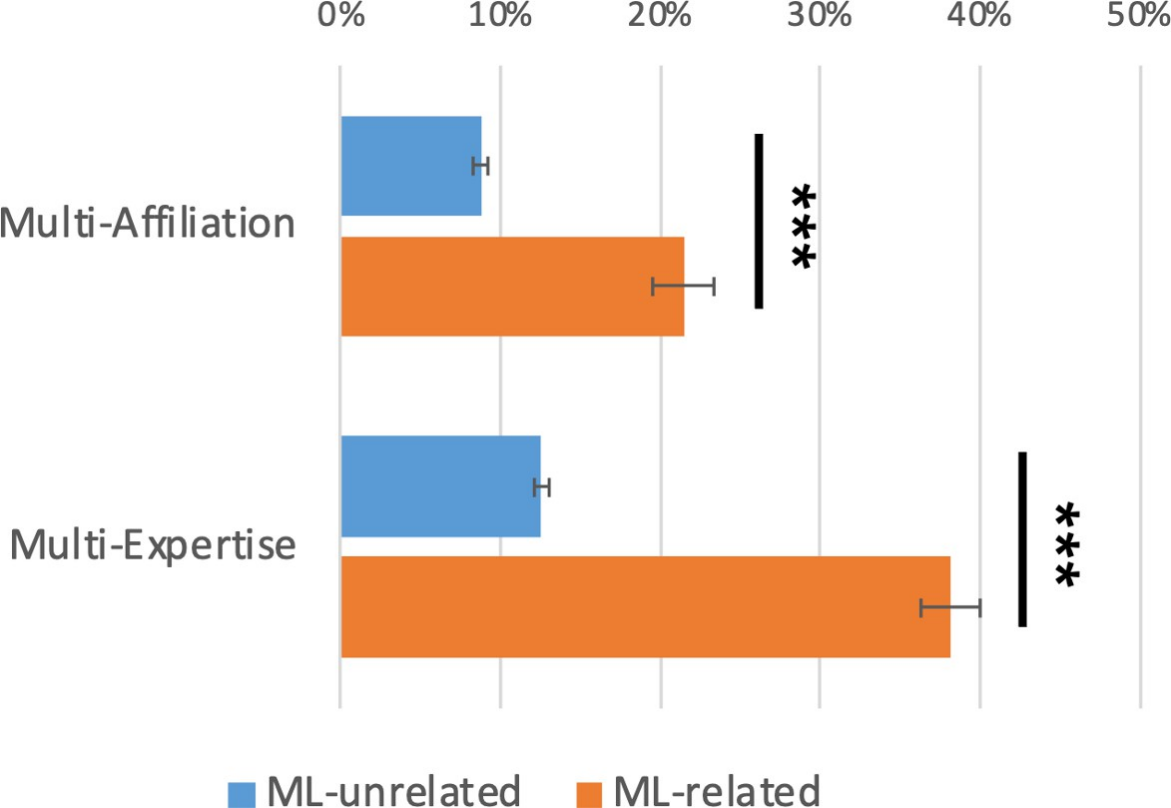
Interdisciplinary expertise. Collaboration forms are estimated by logit regressions controlling for publication years and journals. The error bars indicate one standard error. Two-tailed test: ***p<0.001.

Finally, Fig 3 compares ML-related and unrelated projects in terms of individual team members having both computational and domain expertise. The figure indicates that ML-related projects are more likely to involve one or more individuals who are affiliated with domain and computer departments (21% vs. 9%, p < .001). Similarly, ML-related projects are more likely to engage individuals who had previous experience in computer and domain sciences (38% vs. 13%, p < .001). These results indicate that ML-related projects do incorporate a combination of computational and domain expertise.

### Quality of output from ML-related projects

#### ML-related vs. ML-unrelated projects

We analyze the quality of scientific output produced by ML-related and unrelated projects. We predicted citation impact and novelty by *ML-related* with controlling for the team size and other variables (Table 2A). Model 1 shows a significantly positive coefficient of *ML-related* (b = .478, p < .001), suggesting that ML-related papers tend to receive more citations than ML-unrelated papers. On the other hand, Model 2 finds no significant coefficient of *ML-related* (b = -.004, p >.1), suggesting that ML-related papers do not necessarily present novel discoveries. We further repeated the same set of analyses with a matching approach, in which ML-related papers are compared with ML-unrelated papers published in the same journal in the same year, finding a consistent result (Table 2B).

**Table 2:** Prediction of publication quality: ML-related vs. ML-unrelated projects.

**(A) Base model**				
	Impact		Novelty	
	Model 1		Model 2	
ln(#Author)	.171***	(.010)	.020***	(.005)
ln(#Org)	.013	(.009)	.008^†^	(.004)
ln(#Country)	.079***	(.011)	-.004	(.005)
Univ-Industry Collab	.046*	(.018)	-.043***	(.009)
ML-related	.478***	(.015)	-.004	(.006)
Year dummies	Yes		Yes	
Journal dummies	Yes		Yes	
F stat	499.220***		37.113***	
R2 adjusted	.660		.173	
N	24641		16433	
**(B) Matched Sample**				
	Impact		Novelty	
	Model 1		Model 2	
ln(#Author)	.161***	(.009)	.019***	(.005)
ln(#Org)	.012	(.009)	.011*	(.004)
ln(#Country)	.066***	(.011)	-.005	(.005)
Univ-Industry Collab	.017	(.017)	-0.40***	(.009)
ML-related	.497***	(.016)	-.001	(.008)
F stat	241.507***		155.761***	
R2 adjusted	.828		.797	
N	24405		15938	

Note. Unstandardized coefficients (standard errors in parentheses).

Two-tailed test: ^†^p<0.1,

*p<0.05,

**p<0.01,

***p<0.001.

Ordinary least squares (OLS). (B) ML-related papers are paired with ML-unrelated papers published in the same journal in the same year.

#### Computer-domain collaboration

We then examine how different collaboration forms affect the quality of publications from ML-related projects (Table 3A). First, Models 1–4 use citation impact as the dependent variable. Model 1 suggests that computer-domain collaboration is associated with higher citation impact (b = .113, p < .01). Breaking down such collaboration, Model 2 finds a weakly significant coefficient (b = .089, p < .1) for intraorganizational collaboration and an insignificant coefficient for inter-organizational collaboration (b = .067, p>.1). This is consistent with H1, but the difference between the two forms of collaboration is insignificant, and thus, proximity in collaboration does not seem to play a major role in this context.

**Table 3:** Prediction of publication quality by team structure.

**(A) ML-related Projects Only**																
	Impact								Novelty							
	Model 1		Model 2		Model 3		Model 4		Model 5		Model 6		Model 7		Model 8	
ln(#Author)	.110**	(.037)	.110**	(.037)	.109**	(.037)	.107*	(.042)	-.012	(.013)	-.012	(.013)	-.009	(.013)	-.009	(.015)
ln(#Org)	.004	(.038)	-.001	(.038)	-.002	(.038)	.029	(.041)	.037**	(.013)	.035**	(.013)	.034**	(.013)	.021	(.014)
ln(#Country)	.144***	(.044)	.150***	(.044)	.136**	(.044)	.102*	(.048)	-.003	(.015)	-.001	(.015)	-.003	(.015)	-.000	(.016)
Univ-Industry Collab	.032	(.051)	.033	(.051)	.032	(.051)	.002	(.058)	-.062***	(.018)	-.061***	(.018)	-.063***	(.018)	-.061**	(.020)
Comp-Domain Collab	.113**	(.037)							-.002	(.013)						
Intra-org Collab			.089^†^	(.048)							.012	(.016)				
Inter-org Collab			.067	(.041)							-.006	(.014)				
Multi-Affiliation					.160***	(.046)							.017	(.016)		
Comp-Domain Collab w/o Multi-Affiliation					.056	(.046)							-.021	(.016)		
Multi-Expertise							.119**	(.044)							.026^†^	(.015)
Comp-Domain Collab w/o Multi-Expertise							.139**	(.053)							-.013	(.018)
Year dummies	Yes		Yes		Yes		Yes		Yes		Yes		Yes		Yes	
Journal dummies	Yes		Yes		Yes		Yes		Yes		Yes		Yes		Yes	
F stat	42.377***		41.923***		41.937***		31.402***		6.086***		6.027***		5.820***		5.327***	
R2 adjusted	.611		.611		.606		.587		.184		.184		.176		.189	
N	2530		2530		2505		2034		2137		2137		2117		1724	
**(B) ML-related vs. ML-unrelated Projects**																
	Impact								Novelty							
	Model 1				Model 2				Model 3				Model 4			
ln(#Author)	.170***		(.010)		.159***		(.011)		.021***		(.005)		.025***		(.005)	
ln(#Org)	.007		(.009)		.011		(.010)		.007		(.005)		.003		(.005)	
ln(#Country)	.080***		(.011)		.069***		(.012)		-.003		(.005)		.001		(.006)	
Univ-Industry Collab	.049**		(.018)		.048*		(.020)		-.043***		(.009)		-.040***		(.009)	
ML-related	.457***		(.016)		.460***		(.020)		-.007		(.007)		-.010		(.009)	
Multi-Affiliation (ML-unrelated)	.006		(.017)						.002		(.008)					
Multi-Affiliation (ML-related)	.119***		(.033)						.017		(.014)					
Multi-Expertise (ML-unrelated)					-.006		(.016)						-.006		(.008)	
Multi-Expertise (ML-related)					.006		(.031)						.024^†^		(.013)	
Year dummies	Yes				Yes				Yes				Yes			
Journal dummies	Yes				Yes				Yes				Yes			
F stat	486.705***				412.463***				34.530***				30.739***			
R2 adjusted	.654				.665				.164				.176			
N	24392				20095				16250				13386			

Note. Unstandardized coefficients (standard errors in parentheses).

Two-tailed test: ^†^p<0.1,

*p<0.05,

**p<0.01,

***p<0.001.

Ordinary least squares (OLS). (B) To compare the impact of *Multi-Affiliation* and *Multi-Expertise* between ML-related and ML-unrelated projects, we interacted *Multi-Affiliation* (-*Expertise*) with *ML-related*. For example, *Multi-Affiliation (ML-related)* = 1 if *Multi-Affiliation* = 1 and *ML-related* = 1.

Model 3 further breaks down computer-domain collaboration into ones involving individuals affiliated with both computer and domain departments (*Multi-Affiliation*) and ones not involving such individuals (*Comp-Domain Collab without Multi-Affiliation*), finding that only the former group is associated with higher citation impact (b = .160, p < .001) but not the latter (b = .055, p>.1). This suggests that interdisciplinary individuals are important to achieve high citation impact. Similarly, Model 4 breaks down computer-domain collaboration into ones involving individuals having previous experience in computer and domain sciences (*Multi-Expertise*) and ones not involving such individuals (*Comp-Domain Collab without Multi-Expertise*). The result shows significantly positive coefficients for both variables (b = .119, p < .001 and b = .139, p < .001), suggesting that high citation impact requires either computer-domain collaboration or interdisciplinary individuals. These results are supportive to H2.

Models 5–8 repeat the same set of analyses with novelty as the dependent variable. Model 5 shows that computer-domain collaboration makes no difference in novelty. Model 6 presents a more positive coefficient for intra-organizational collaboration than for inter-organizational collaboration (b = .012 vs. b = -.006), which is consistent with H1, but they are both insignificant. Finally, Model 8 finds that interdisciplinary individuals having both computer and domain science expertise are weakly associated with higher novelty (b = .026, p < .1), consistent with H2.

These results seem to imply relatively greater importance of interdisciplinary individuals rather than interdisciplinary collaboration. Thus, we further test whether the role of interdisciplinary individuals is specific to ML-related projects. To this end, we compare the impact of *Multi-Affiliation* and *Multi-Expertise* between ML-related and ML-unrelated projects (Table 3B). In all four models, we find that the coefficients are larger for ML-related projects than for ML-unrelated projects. In particular, Model 1 shows that individuals having both computer and domain affiliations (*Multi-Affiliation*) are significantly associated with higher citation impact (b = .119, p < .001), and Model 4 shows that individuals having both computer and domain expertise (*Multi-Expertise*) are significantly associated with higher novelty (b = .024, p < .1).

#### Interdependency of computer and domain science

Next, we test whether the role of computer-domain collaboration and interdisciplinary individuals differs due to the interdependency of the two expertise areas. To this end, Table 4 draws on the subsamples of ML-related projects–*computation-focused* projects and *computer-domain integrated* projects. We first test whether the proximity of collaboration shows a different impact between the two subsamples, finding mostly insignificant results. Thus, H3A is rejected. We then test whether interdisciplinary individuals make a greater impact on interdependent projects (Table 4). The result indeed shows that both *Multi-Affiliation* and *Multi-Expertise* have significantly positive coefficients only in computer-domain integrated projects. This suggests that interdisciplinary individuals are particularly important when projects employ both computational and domain approaches interdependently, supporting H3B.

**Table 4:** Use of machine: Computation-focused vs. computer-domain integrated projects (ML-related projects only).

	Impact								Novelty							
	Computation-focused				Computer-Domain Integrated				Computation-focused				Computer-Domain Integrated			
	Model 1		Model 2		Model 3		Model 4		Model 5		Model 6		Model 7		Model 8	
ln(#Author)	.008	(.082)	.014	(.099)	.112	(.071)	.117	(.080)	-.017	(.031)	-.018	(.037)	-.004	(.023)	.001	(.026)
ln(#Org)	-.030	(.087)	.036	(.094)	-.015	(.068)	.047	(.073)	-.004	(.032)	-.016	(.034)	-.029	(.022)	-.021	(.024)
ln(#Country)	.157^†^	(.095)	.162	(.108)	.172*	(.078)	.112	(.084)	.018	(.034)	.038	(.038)	.049^†^	(.025)	.047^†^	(.027)
Univ-Industry Collab	.118	(.120)	.063	(.145)	-.079	(.090)	-.109	(.098)	.032	(.047)	.012	(.054)	-.078**	(.029)	-.061^†^	(.031)
Multi-Affiliation	.085	(.098)			.186*	(.074)			.039	(.036)			.058*	(.024)		
Multi-Expertise			.008	(.089)			.164*	(.074)			-.013	(.032)			.063**	(.024)
Year dummies	Yes		Yes		Yes		Yes		Yes		Yes		Yes		Yes	
Journal dummies	Yes		Yes		Yes		Yes		Yes		Yes		Yes		Yes	
F stat	19.116***		11.190***		21.415***		18.534***		2.857***		2.735***		3.492***		3.230***	
R2 adjusted	.611		.532		.665		.662		.160		.186		.211		.217	
N	613		476		751		646		487		380		664		572	

Note. Unstandardized coefficients (standard errors in parentheses).

Two-tailed test: ^†^p<0.1,

*p<0.05,

**p<0.01,

***p<0.001.

Ordinary least squares (OLS).

#### Depth of ML

Finally, we break down ML technologies. First, Table 5A regresses the publication quality on *DL-related* in addition to *ML-related*. Model 1 shows that DL-related papers are even more cited compared to DL-unrelated papers (b = .200, p < .001). However, Model 2 indicates that DL-related papers are less novel compared to DL-unrelated papers (b = -.054, p < .001). Thus, it appears that DL does not necessarily allow scientists to gain novel insights beyond human cognition. DL rather seems to be applied to an agenda that humans have relatively good understanding of (and thus with lower novelty). Our interviewee suggested that DL tends to provide better model performance (e.g., accuracy, precision), facilitating further uses of DL models, which is consistent with the positive coefficient of *DL-related* on citation count (Model 1).

**Table 5:** ML technologies.

**(A) Base model**															
	Impact								Novelty						
	Model 1								Model 2						
ln(#Author)	.170***				(.010)				.021***				(.005)		
ln(#Org)	.014				(.009)				.007^†^				(.004)		
ln(#Country)	.079***				(.011)				-.003				(.005)		
Univ-Industry Collab	.045*				(.018)				-.043***				(.009)		
ML-related	.401***				(.018)				.017*				(.008)		
DL-related	.200***				(.028)				-.054***				(.012)		
Year dummies	Yes								Yes						
Journal dummies	Yes								Yes						
F stat	495.598***								36.987***						
R2 adjusted	.661								.174						
N	24641								16433						
**(B) Interdisciplinary Expertise (ML-related projects only)**															
	Computation-focused				Computer-Domain Integrated				Computation-focused				Computer-Domain Integrated		
	Model 1		Model 2		Model 3		Model 4		Model 5		Model 6		Model 7		Model 8
ln(#Author)	-.014	(.082)	-.003	(.097)	.114	(.071)	.112	(.080)	-.013	(.031)	-.013	(.037)	(.023)	.003	(.026)
ln(#Org)	-.021	(.086)	.027	(.093)	-.011	(.069)	.055	(.073)	-.004	(.031)	-.015	(.034)	(.022)	-.024	(.024)
ln(#Country)	.168^†^	(.094)	.179^†^	(.106)	.173*	(.078)	.110	(.084)	.016	(.033)	.035	(.038)	(.025)	.046^†^	(.027)
Univ-Industry Collab	.100	(.119)	.066	(.143)	-.088	(.090)	-.124	(.099)	.031	(.046)	.004	(.055)	(.029)	-.052^†^	(.032)
DL-related	.234**	(.079)	.198^†^	(.109)	.090	(.084)	.121	(.098)	-.023	(.029)	-.026	(.040)	(.027)	-.065*	(.032)
Multi-Affiliation (DL-unrelated)	-.012	(.137)			.201*	(.085)			.100^†^	(.052)			(.027)		
Multi-Affiliation (DL-related)	.113	(.124)			.127	(.135)			.003	(.046)			(.044)		
Multi-Expertise (DL-unrelated)			-.135	(.126)			.214*	(.086)			.005	(.047)		.042	(.028)
Multi-Expertise (DL-related)			.102	(.114)			.031	(.133)			-.026	(.040)		.118**	(.043)
Year dummies	Yes		Yes		Yes		Yes		Yes		Yes			Yes	
Journal dummies	Yes		Yes		Yes		Yes		Yes		Yes			Yes	
F stat	19.029***		11.414***		20.834***		18.050***		2.845***		2.667***		3.444***		3.212***
R2 adjusted	.618		.547		.665		.662		.165		.186		.212		.220
N	613		476		751		646		487		380		664		572

Note. Unstandardized coefficients (standard errors in parentheses).

Two-tailed test: ^†^p<0.1,

*p<0.05,

**p<0.01,

***p<0.001.

Ordinary least squares (OLS).

Table 5B further investigates the contribution of interdisciplinary individuals in DL-related and DL-unrelated projects by distinguishing computation-focused projects and computer-domain integrated projects. The result shows almost no effect of interdisciplinary individuals in computation-focused projects (Models 1, 2, 5, and 6), as in Table 4. In contrast, in computer-domain integrated projects, interdisciplinary individuals seem to play different roles in DL-related and DL-unrelated projects. In terms of citation impact, Models 3 and 4 show that interdisciplinary individuals are more important in DL-unrelated projects (but ML-related) than in DL-related projects. On the other hand, in terms of novelty, Models 5 and 6 show that interdisciplinary individuals are more important in DL-related projects than in DL-unrelated ones. A plausible interpretation is that greater novelty is rooted in inspiration from DL facilitated by the integration of computer and domain expertise, whereas greater citation results from higher model performance due to DL, which may not necessarily require the fundamental integration of computer and domain expertise.

## Discussion and conclusion

The progress of science increasingly relies on computational expertise and particularly on ML [6], and machines work alongside humans in various domains. The integration of machines as creative agents in science can influence the optimal design of work and organizations [11–13]. This study thus investigated the team structure of ML-related projects and analyzed the contribution of ML to scientific knowledge production under different team structure, drawing on bibliometric data of 25,000 scientific publications in six scientific domains.

This study contributes to the literature by illustrating the role of machines in a scientist team. Previous literature on the use of machines has been either at a macro level [6] or at a micro (cognitive) level [4], with the meso-level discussion in scientist teams remaining to be understudied [14]. Although a few recent studies described the patterns of collaboration (e.g., international vs. domestic collaboration) in ML-related projects [15, 16], our understanding has been scarce as to how ML affects the quality of scientific knowledge production under different internal team structures. Drawing on the literature of scientific collaboration and interdisciplinarity [28, 29, 36, 49], we argue that team features that help integrate computer expertise and domain expertise are associated with higher output quality, and that these features are more important when the computer-related tasks and domain tasks are interdependent.

A potential challenge in integrating computer and domain sciences is the lack of incentive. While our analysis shows that interdisciplinarity contributes to higher impacts and novelty, it is not obvious whether scientists with computational expertise and those with domain expertise are willing to work together. In fact, our interview suggested that scientists working on ML tended to appreciate publications in computer science rather than publications in other fields. It is also suggested that domain scientists do not always appreciate research approaches based upon ML because it is difficult to explain how prediction is made by a model that could be considered a black box. It is thus critical to understand what motivates ML scientists to collaborate with domain scientists and what obstacles exist in their collaboration.

It is particularly interesting to find that ML can contribute to the *novelty* of scientific discoveries with the engagement of interdisciplinary individuals. Our empirical work further suggests that interdisciplinary individuals are critical in delivering novel discoveries based on deeper ML (DL). ML can bring various values, such as the efficiency of data analysis or greater precision of model prediction. However, supplementing humans’ cognitive capacity and delivering novel discoveries is a fundamental benefit of machines under the burden of knowledge [31]. Our result highlights a critical role played by interdisciplinary individuals in achieving novelty. Thanks to the continued advancement of computational science, more sophisticated and potentially more complex and deeper ML approaches are likely to become available for domain scientists. Our result implies that engaging interdisciplinary individuals is crucial in exploiting the full capacity of computational techniques. Indeed, our interviewee referred to a critical "liaison" role played by a scientist who studied computer science and genetics in a project utilizing ML for detecting cancers. Such interdisciplinary scientists may begin their careers either as domain scientists or as computer scientists. For example, domain sciences have invested in computational techniques (e.g., bioinformatics) to systematically train interdisciplinary scientists [47]. Efforts may need to be further reinforced, for example, to support educational programs to nurture interdisciplinary scientists who can integrate computer and domain sciences.

Our results need to be interpreted with a few limitations. First, our bibliometric approach captures the structure of scientist teams only partially. Future research should look into how team members interact and what skills each member has. Second, future research should further investigate the technical aspects of ML. ML can be based on various models and can differ particularly in depth and complexity. We attempted to capture the depth by a simplified approach, but the result needs careful interpretation and a more sophisticated analysis is required. Third, we have to be cautious about potential changes in the role of machines over time. The vast majority of our sampled papers were published in the last four years (2017–2021) because ML is a rather recent phenomenon. The role of machines and how it affects the team design might change in the future with the advancement of computational techniques. Fourth, our results are based on cross-sectional analyses, and thus, the causal mechanism behind our findings cannot be completely clear.

## Supporting information

S1 File The dataset underlying the results. (ZIP)

S1 Appendix Selection of fields and journals. (PDF)
