# Supplementary material for: Role of machine and organizational structure in science
Source: PLoS One. 2022 Aug 11;17(8):e0272280. doi: 10.1371/journal.pone.0272280 (PMC9371286; doi:10.1371/journal.pone.0272280)
Supplement: S1 Appendix — (PDF) [file pone.0272280.s002.pdf]

S2 Appendix. Selection of Fields and Journals

Field	Subject category	Journal 1	Journal 2	Journal 3	Journal 4	Journal 5
Agriculture	Food Science & Technology	Foods	LWT Food Science and Technology	Food Research International	International Journal of Food Science and Technology	Food Control
	Plant Sciences	Frontiers in Plant Science	Plants Basel	New Phytologist	Plant Disease	Journal of Experimental Botany
Biology	Biochemistry & Molecular Biology	Biomolecules [34]	Journal of Biological Chemistry	Nucleic Acids Research	FEBS Journal	Metabolites
	Neurosciences	Frontiers in Neuroscience	Brain Sciences	Journal of Neuroscience	Journal of Alzheimers Disease	Neuroscience
	Biotechnology & Applied Microbiology	Applied Microbiology and Biotechnology	Applied and Environmental Microbiology	Journal of Applied Microbiology	Biotechnology and Bioengineering	Nature Biotechnology
	Cell Biology	Cells	Cell Reports	Cell Death & Disease	Oxidative Medicine and Cellular Longevity	Journal of Cell Science
	Biology	ELife	Journal of Experimental Biology	Biology-Basel	Saudi Journal of Biological Sciences	Philosophical Transactions of the Royal Society B-Biological Sciences
	Immunology	Frontiers in Immunology	Journal of Immunology	Journal of Clinical Immunology	Cellular Molecular Immunology	Nature Immunology
Chemistry	Chemistry, Physical	Catalysts	ACS Catalysis	Colloids and Surfaces A Physicochemical and Engineering Aspects	Journal of Colloid and Interface Science	Journal of Physical Chemistry B
	Chemistry, Multidisciplinary	RSC Advances	Angewandte Chemie International Edition	ASC Omega	Chemical Communications	Journal of The American Chemical Society
	Chemistry, Organic	Organic Letters	Journal of Organic Chemistry	Organic Biomolecular Chemistry	European Journal of Organic Chemistry	Tetrahedron Letters
Material Sciences	Materials Science, Multidisciplinary	Materials	Journal of Materials Science	Materials Chemistry and Physics	Materials Today Communications	Materials Design
Medicine	Pharmacology & Pharmacy	Frontiers in Pharmacology	European Review for Medical and Pharmacological Sciences	Pharmaceutics	International Journal of Pharmaceutics	Clinical Pharmacology Therapeutics
	Surgery	British Journal of Surgery	Journal of The American College of Surgeons	Surgical Endoscopy and Other Interventional Techniques	Obesity Surgery	Plastic and Reconstructive Surgery
	Oncology	Journal of Clinical Oncology	Cancers	Annals of Oncology	Frontiers in Oncology	Cancer Research
	Clinical Neurology	Neurology	Movement Disorders	Journal of Neurology	Parkinsonism Related Disorders	Multiple Sclerosis and Related Disorders
	Medicine, General & Internal	Journal of Clinical Medicine	BMJ British Medical Journal	BMJ Open	Jama Journal of the American Medical Association	New England Journal of Medicine
Physics	Physics, Applied	Journal of Applied Physics	Applied Physics Letters	Journal of Physics D Applied Physics	Physical Review Applied	Applied Physics Express
	Physics, Condensed Matter	Journal of Physics Condensed Matter	Annual Review of Condensed Matter Physics	Solid State Physics	Advances in Physics	
	Physics, Multidisciplinary	Physical Review Letters	Entropy	Physica A Statistical Mechanics and Its Applications	European Physical Journal Plus	Physica Scripta

Note. In each SC, we selected up to five journals that are associated with only a single SC. We further selected only Tier-1 and Tier-2 journals in the WoS journal ranking. In one SC (Physics, Condensed Matter), we found only four journals that satisfy the conditions. Thus, we selected 99 journals in total.
